# Pulmonary Cystic Echinococcosis

**DOI:** 10.4269/ajtmh.17-0298

**Published:** 2017-09-07

**Authors:** Xavier Argemi, Nicola Santelmo, Nicolas Lefebvre

**Affiliations:** 1 Hôpitaux Universitaires, Maladies Infectieuses et Tropicales, Strasbourg, France;; 2 Hôpitaux Universitaires, Chirurgie Thoracique, Strasbourg, France

## CASE REPORT

**Figure 1.**
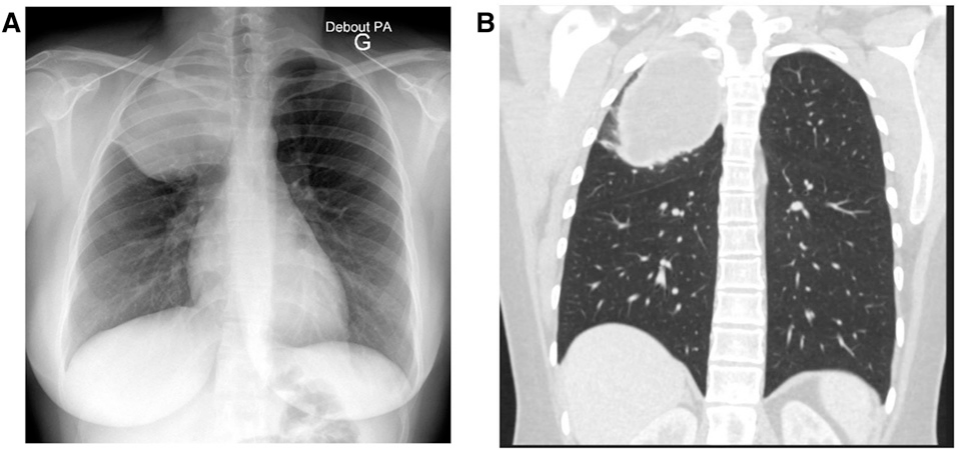
Chest radiography (Panel **A**) and computed tomography scan (Panel **B**) showing a cyst-like mass with smooth borders and no calcification in a Turkish woman presenting with a 1-month history of chest pain.

**Figure 2.**
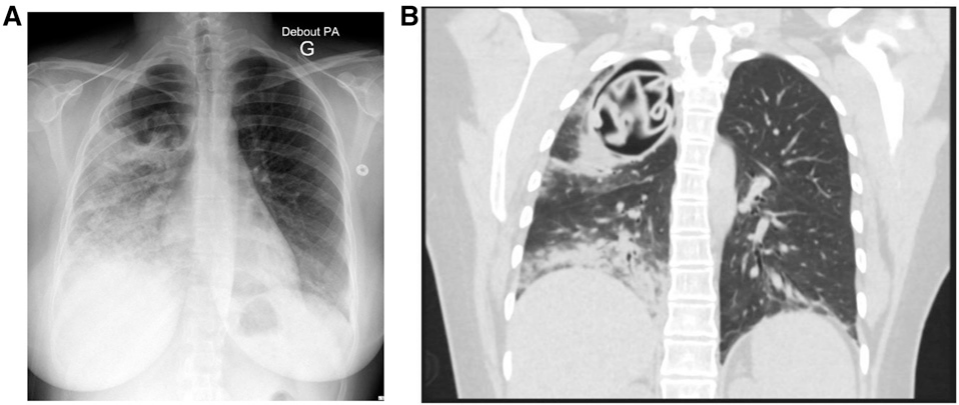
Chest radiography (Panel **A**) and computed tomography scan (Panel **B**) showing a ruptured hydatid cyst with underneath interstitial pneumonia and apparition of a pathognomonic germinative membrane.

A 27-year-old Macedonian woman, coming from Turkey and living in France since 2012, presented with a 1-month history of chest pain. This housewife had no medical history, no animal exposure, and used to live in small villages. At admission, she was afebrile, physical examination revealed reduced vesicular breath sounds of the right upper lobe. Chest radiography and whole body computed tomography scan showed a thoracic cyst-like mass with smooth borders, no calcification, and ruled out intraabdominal lesion (Figure 1A and B). *Echinococcus granulosus* infection was suggested due to positive Western Blot against p7 and p26-kD bands. A lobectomy was planned 1 month later, and the patient went back home. She came back 3 weeks later for sudden onset of chest pain, cough, fever with eosinophilia (3,270 mm^3^), and elevated C-reactive protein level (226 mg per litter). The rupture of the cyst with underneath interstitial pneumonia was established (Figure 2A and B). Lobectomy was performed 1 week after antiparasitic drug therapy with albendazole. Surgery evidenced an empty cyst that were totally removed and a bronchial fistula that were sutured. Pathology analysis confirmed the presence of *Echinococcus granulosus* protoscolex and rostellar hooks. Albendazole 15 mg/kg per day was continued for 6 months. After 3-years follow-up, there was no evidence of relapse and chest radiography was normal.

## DISCUSSION

Echinococcosis is a cestodiasis caused by infection with the larval stage of *Echinococcus*. Four species of *Echinococcus* are described in humans: *E. granulosus* causing cystic echinococcosis (CE), and *Echinococcus multilocularis* that lead to alveolar echinococcosis, are the most common.^1^ The two other species, *Echinococcus vogeli* and *Echinococcus oligarthrus*, cause polycystic echinococcosis but have been rarely described in human infections. *E. granulosus* is the most frequent cause of the disease resulting in unilocular cystic lesions usually located in the liver and the lung but that may affect all other organs. Although most infections are asymptomatic, CE causes slowly enlarging cysts that often grow unnoticed and neglected for years until acute complications occur as cyst rupture producing cough, chest pain, hemoptysis, or vomica. Surgery is still the main therapeutic option to remove the cyst and suture bronchial fistula if necessary, associated with prolonged antiparasitic therapy.^2^ The world health organization has developed guidelines based on the available evidence than can assist in determining appropriate treatment strategies.^3^
